# Association between circulating vitamin E and ten common cancers: evidence from large-scale Mendelian randomization analysis and a longitudinal cohort study

**DOI:** 10.1186/s12916-022-02366-5

**Published:** 2022-05-11

**Authors:** Junyi Xin, Xia Jiang, Shuai Ben, Qianyu Yuan, Li Su, Zhengdong Zhang, David C. Christiani, Mulong Du, Meilin Wang

**Affiliations:** 1 Jiangsu Cancer Hospital, Jiangsu Institute of Cancer Research, The Affiliated Cancer Hospital of Nanjing Medical University, Nanjing, China; 2 Department of Environmental Genomics, Jiangsu Key Laboratory of Cancer Biomarkers, Prevention and Treatment, Collaborative Innovation Center for Cancer Personalized Medicine, Center for Global Health, School of Public Health, Nanjing Medical University, 101 Longmian Avenue, Jiangning District, Nanjing, 211166 China; 3 Department of Clinical Neuroscience, Center for Molecular Medicine, Karolinska Institutet, Stockholm, Sweden; 4 Department of Environmental Health, Harvard T.H. Chan School of Public Health, Boston, MA USA; 5 Department of Medicine, Massachusetts General Hospital, Boston, MA USA; 6 Department of Biostatistics, Center for Global Health, School of Public Health, Nanjing Medical University, 101 Longmian Avenue, Jiangning District, Nanjing, 211166 China; 7 The Affiliated Suzhou Hospital of Nanjing Medical University, Suzhou Municipal Hospital, Gusu School, Nanjing Medical University, Suzhou, China

**Keywords:** Circulating vitamin E, Cancer risk, Mendelian randomization, GWAS, UK Biobank

## Abstract

**Background:**

The association between vitamin E and cancer risk has been widely investigated by observational studies, but the findings remain inconclusive. Here, we aimed to evaluate the causal effect of circulating vitamin E on the risk of ten common cancers, including bladder, breast, colorectal, esophagus, lung, oral and pharynx, ovarian, pancreatic, prostate, and kidney cancer.

**Methods:**

A Mendelian randomization (MR) analytic framework was applied to data from a cancer-specific genome-wide association study (GWAS) comprising a total of 297,699 cancer cases and 304,736 controls of European ancestry. Three genetic instrumental variables associated with circulating vitamin E were selected. Summary statistic-based methods of inverse variance weighting (IVW) and likelihood-based approach, as well as the individual genotyping-based method of genetic risk score (GRS) were used. Multivariable IVW analysis was further performed to control for potential confounding effects. Furthermore, the UK Biobank cohort was used as external validation, supporting 355,543 European participants (incident cases ranged from 437 for ovarian cancer to 4882 for prostate cancer) for GRS-based estimation of circulating vitamin E, accompanied by a one-sample MR analysis of dietary vitamin E intake underlying the time-to-event analytic framework.

**Results:**

Specific to cancer GWAS, we found that circulating vitamin E was significantly associated with increased bladder cancer risk (odds ratios [OR]_IVW_ = 6.23, *P*_IVW_ = 3.05×10^-3^) but decreased breast cancer risk (OR_IVW_ = 0.68, *P*_IVW_ = 8.19×10^-3^); however, the significance of breast cancer was dampened (*P*_multivariable IVW_ > 0.05) in the subsequent multivariable MR analysis. In the validation stage of the UK Biobank cohort, we did not replicate convincing causal effects of genetically predicted circulating vitamin E concentrations and dietary vitamin E intake on the risk of ten cancers.

**Conclusions:**

This large-scale population study upon data from cancer-specific GWAS and a longitudinal biobank cohort indicates plausible non-causal associations between circulating vitamin E and ten common cancers in the European populations. Further studies regarding ancestral diversity are warranted to validate such causal associations.

**Supplementary Information:**

The online version contains supplementary material available at 10.1186/s12916-022-02366-5.

## Background

Vitamin E is a group of fat-soluble antioxidant nutrients consisting of tocopherols and tocotrienols. Tocopherol, a major isoform of vitamin E, has been found to eliminate reactive oxygen species, inhibit carcinogenesis and tumor growth, and stimulate cancer cell apoptosis [1, 2].

Albeit the associations between vitamin E and cancer risk have been explored by several epidemiological studies, their findings remain inconsistent [3]. For instance, the Selenium and Vitamin E Cancer Prevention Trial (SELECT) found that supplementation with vitamin E was associated with an increased risk of prostate cancer among 34,887 men [4], but this was not confirmed in the Physicians’ Health Study II randomized trial following 14,641 men [5]. Although randomized trials are commonly recognized as the gold standard for making causal inferences, they are usually not widely available due to high cost and long duration. Nevertheless, even randomized trials are likely to be underpowered given the low incidence of endpoint phenotypes such as rare cancers [6].

Mendelian randomization (MR), a novel statistical approach that uses genetic variants associated with exposure of interest as instruments, can be applied to estimate a causal relationship between exposure and outcome [7]. MR is designed based on the fact that genetic variants are randomly allocated during gamete formation and conception, therefore independent of confounding factors. Results from MR designs are thus less susceptible to reverse causality and confounding bias [8]. In this study, we leveraged large-scale genome-wide genetic data and UK Biobank cohort of European ancestry to apply an MR framework, to estimate a putative causal association of circulating vitamin E with the risk of ten common cancers (Additional file 1: Fig. S1).

## Methods

### Study subjects

#### Cancer-specific case-control genome-wide association studies (GWASs)

The current MR analysis was comprehensively performed by leveraging information from ten GWASs totaling 602,435 participants of European ancestry, including 297,699 cancer cases and 304,736 controls across the bladder, breast, colorectal, esophagus, lung, oral and pharynx, ovarian, pancreatic, prostate, and kidney cancer. The characteristics of each cancer-specific GWAS including sample sizes and data sources are illustrated in Additional file 1: Table S1.

Briefly, as outcomes of interest, we collected available GWAS data across ten cancers. For summary-level GWAS data of 4 cancers (i.e., breast, ovarian, prostate, and lung cancer), quality control procedures and population details have been described elsewhere [9–12]. For six cancers (bladder, colorectal, esophagus, oral and pharynx, pancreatic, and kidney cancer) which we had access to individual-level genotyping data [13–23], we performed stringent quality control procedures of population via removing unexpected duplicates or probable relatives based on pairwise identity by descent, guaranteeing all individuals to be of European ancestry.

#### UK Biobank cohort data

The UK Biobank cohort was a prospective population-based study that recruited 502,528 adults aged 40–69 years from the general population between April 2006 and December 2010. The study protocol and information about data access are available online (http://www.ukbiobank.ac.uk/), and more details of the recruitment and study design have been published in previous studies [24]. The UK Biobank resource used by this study was under Application #45611.

After the quality control of the following population: (i) excluded individuals with prevalent cancer (except non-melanoma skin cancer, based on the International Classification of Diseases, 10th revision [ICD-10, C44]) at baseline; (ii) excluded individuals of sex discordance; (iii) excluded outliers for genotype missingness or excess heterozygosity; (iv) retained unrelated participants; (v) restricted to “white British” individuals of European ancestry; and (vi) removed individuals who decided not to participate in this program, a total of 355,543 participants remained for analysis. Moreover, we defined the ten cancers using the ICD-10 codes (Additional file 1: Table S2). The follow-up time was calculated from baseline assessment to the first diagnosis of cancer, loss to follow-up, death, or last follow-up (December 14, 2016), whichever occurred first.

Information on dietary vitamin E intake in UK Biobank participants was retrieved from data field #100025 (description: vitamin E; category: estimated nutrients yesterday—diet by a 24-h recall—online follow-up). Measurements were performed at baseline (2006–2010) and/or subsequent follow-up visits. In the present study, we included 49,579 individuals (23,107 males and 26,472 females) with baseline vitamin E measurements.

### Two-sample MR analysis and sensitivity analysis of cancer-specific GWAS

Based on cancer-specific GWAS databases, depends on the availability of data, we applied a summary statistics-based approach to all cancers, and additionally, a genetic risk score (GRS)-based approach to some of the cancers (bladder, colorectal, esophagus, oral and pharynx, pancreatic, and kidney cancer), followed by sensitivity analysis.

#### Instrumental variable (IV) selection

Circulating vitamin E was the main exposure of interest. We collected 3 independent GWAS-identified circulating vitamin E-associated single-nucleotide polymorphisms (SNPs; rs964184, rs11057830, and rs2108622) from a large GWAS available to date [25], which met the following criteria as instruments for MR analysis: (i) reported *P*-value < 5.00×10^-8^, (ii) minor allele frequency (MAF) ≥ 0.05, (iii) call rate ≥ 95%, and (iv) Hardy-Weinberg equilibrium (HWE) *P*-value in controls ≥ 1×10^-6^ (Additional file 1: Table S3). The online web tool mRnd (https://cnsgenomics.shinyapps.io/mRnd/) was used to estimate statistical power [26]. To calculate the minimum detectable effect size, we set 80.0% statistical power and 5.0% alpha level and used the proportion of circulating vitamin E variance (*R*^*2*^, i.e., 1.7% estimated by Major et al.) explained by the 3 IVs as calculated in the previous GWAS [25, 27]. We further quantified the strength of IVs by F-statistics, where F-statistics > 10 provided good evidence for the IV being a strong instrument [28].

#### Summary statistic-based method

The summary statistics-based methods, including an inverse variance weighting (IVW) method and a likelihood-based method, were primarily used to infer causal associations. The formula of IVW method was as follows: ${\beta}_{IVW}=\frac{\sum_{i=1}^k{\beta}_{Xi}{\beta}_{Yi}{\sigma}_{Yi}^{-2}}{\sum_{i=1}^k{\beta}_{Xi}^2{\sigma}_{Yi}^{-2}}$; ${SE}_{IVW}=\sqrt{\frac{1}{\sum_{i=1}^k{\beta}_{Xi}^2{\sigma}_{Yi}^{-2}}}$, where *i* is the *i*th SNP, *β*_X*i*_, and σ_X*i*_ are the estimate and standard error of genetic association with the exposure that were derived from IVs, and *β*_Y*i*_ and σ_Y*i*_ are the estimate and standard error of genetic association with the outcome that were derived from cancer-specific GWAS. In addition, we adopted the likelihood-based method, which can be used to obtain appropriately sized confidence intervals when there is considerable imprecision in the estimates.

#### GRS-based method

We further constructed a weighted GRS to integrate the genetic effects of candidate SNPs on the exposure of interest for available individual-level genotyping data. We summed three circulating vitamin E-associated SNPs weighted by corresponding effect sizes using the formula: $\mathrm{GRS}={\sum}_{i=1}^n{\beta}_i{\mathrm{SNP}}_{\mathrm{i}}$, where *n* is the number of SNPs, SNP_*i*_ is the number of risk alleles (0, 1, 2) carried by the *i*th SNP, and *β*_*i*_ is the previously published regression coefficient for *i*th SNP. We then evaluated the association of circulating vitamin E-GRS with cancer risk through the logistic regression model with adjustment for sex, age, and the first ten principal components when appropriate.

Multiple testing correction was performed by false discovery rate (FDR) method using the “p.adjust” function in R software.

#### Sensitivity analysis

Estimates from MR can only be reliably interpreted when three model assumptions are valid, including (i) the IVs are associated with exposure variables, (ii) the IVs are not related to other confounding factors, and (iii) the IVs only influence outcome variables through their effects on exposure variables. Therefore, we performed heterogeneity analysis and MR-Egger regression analysis to evaluate the potential violation to the second and third assumptions. The heterogeneity test was used to assess whether a genetic variant’s effect on cancer risk was proportional to its effect on circulating vitamin E. MR-Egger regression (MR-Egger intercept test) was fitted to evaluate the presence of horizontal pleiotropy. We additionally conducted a leave-one-out analysis where we excluded one SNP at a time and performed IVW analysis on the remaining two SNPs to evaluate the robustness of our results.

Furthermore, to control for the effects of potential confounding factors on significant associations of univariable MR analyses, we also conducted multivariable IVW analysis using the effect size retrieved from the Gene ATLAS database (http://geneatlas.roslin.ed.ac.uk/) [29].

### Validation in the UK Biobank cohort

#### Circulating vitamin E based GRS analysis

We used Cox proportional hazards models to calculate hazard ratios (HRs) and 95% confidence intervals (CIs) for the associations between circulating vitamin E-GRS and the risk of ten cancers, with the adjustment of sex, age, study centers, body mass index (BMI), smoking status, drinking status, and first ten principal components when appropriate.

#### One-sample MR analysis for dietary vitamin E intake

One-sample MR analysis was used to evaluate the association between dietary vitamin E intake at baseline and the cancer risk. The genetic IVs for one sample MR were extracted from the UK Biobank imputation dataset, which followed the extensive quality control of SNPs, including (i) imputation confidence score (info score) ≥ 0.3, (ii) MAF ≥ 0.05, (iii) call rate ≥ 95%, and (iv) HWE *P*-value ≥ 1×10^-6^. Then, we performed linear regression analysis between each variant and log-transformed dietary vitamin E measurements, to provide independent (linkage disequilibrium *r*^*2*^ < 0.1) dietary vitamin E-associated IVs under different significance thresholds (i.e., *P*-value ≤ 5.00×10^-7^, *P*-value ≤ 5.00×10^-6^, *P*-value ≤ 5.00×10^-5^). These IVs with different significance thresholds were further used to construct weighted GRS, as well as unweighted GRS to avoid potential over-fitting. In addition, we also annotated the dietary vitamin E-associated lead loci with functional activity (with HaploReg v4.1, https://pubs.broadinstitute.org/mammals/haploreg/haploreg.php) [30] and expression quantitative trait loci (eQTL) analysis (with eQTLGen consortium of 31,684 blood samples, https://www.eqtlgen.org/cis-eqtls.html) [31].

Briefly, a two-stage method was implemented for one-sample MR analysis: (i) the first-stage model consisted of a linear regression of the log-transformed dietary vitamin E measurements on the weighted and unweighted GRSs and (ii) the second-stage model consisted of a Cox regression of the cancer risk on the fitted values from the first-stage optimal regression model (with the strongest correlation with observed dietary vitamin E level). The covariates included sex, age, study centers, BMI, smoking status, drinking status, and the first ten principal components when appropriate.

#### Sensitivity analysis

Several sensitivity analyses were also performed in the UK Biobank cohort, including (i) re-analyzed the association using logistic regression model with incident and prevalent cancer cases in a case-control design and (ii) additionally adjusted for socioeconomic (i.e., education and employment status) and chronic disease status (i.e., coronary artery disease, stroke, hypertension, and type 2 diabetes).

All statistical analyses were performed using R version 3.6.1, and a two-sided *P*-value less than 0.05 was considered as strong evidence for a causal association.

## Results

### Power analysis and genetic effect estimation

For each cancer-specific GWAS, the F-statistics of the 3 IVs are summarized in Table 1. The smallest F-statistic was 72.48 (larger than 10), indicating a strong instrumental strength. In general, our MR analyses obtained sufficient power—we had 80% power to detect moderate effect sizes, with odds ratios (ORs) ranging from 0.44 (kidney cancer) to 0.91 (breast cancer) per standard deviation (SD) increase in circulating vitamin E levels.

**Table 1:** Statistical power in Mendelian randomization (MR) study of circulating vitamin E and cancer risk in cancer-specific GWAS

Cancer type	Sample size (*N* = 602,435)		F-statistics	Minimum detectable OR^a^
	Cases (*N* = 297,699)	Controls (*N* = 304,736)		
Bladder cancer	5930	5468	198.12	0.67/1.49
Breast cancer	122,977	105,974	3960.48	0.91/1.09
Colorectal cancer	24,476	23,073	823.31	0.82/1.22
Esophagus cancer	2268	1865	72.48	0.53/1.97
Lung cancer	29,266	56,450	1483.37	0.85/1.16
Oral and pharynx cancer	4950	2907	136.88	0.63/1.70
Ovarian cancer	22,406	40,941	1096.52	0.83/1.19
Pancreatic cancer	4970	3532	148.03	0.64/1.63
Prostate cancer	79,148	61,106	2426.55	0.89/1.12
Kidney cancer	1308	3420	82.77	0.44/1.81

^a^Minimum detectable OR (per 1 SD of vitamin E) was calculated based on 80% power, 5% alpha level, and 1.7% of vitamin E variance (*R*^*2*^) explained by 3 SNPs used in this study

We next evaluated the genetic effects of each circulating vitamin E-associated SNP on cancer risk and observed that no SNPs were significantly associated with any cancer risk (Additional file 1: Table S4), except for a marginal risk effect of rs964184 on breast cancer (OR = 0.98, *P* = 0.043); as well as rs11057830 (OR = 1.10, *P* = 0.015) and rs2108622 (OR = 1.08, *P* = 0.013) on bladder cancer.

### Causal association between circulating vitamin E and cancer risk

**Fig. 1:**
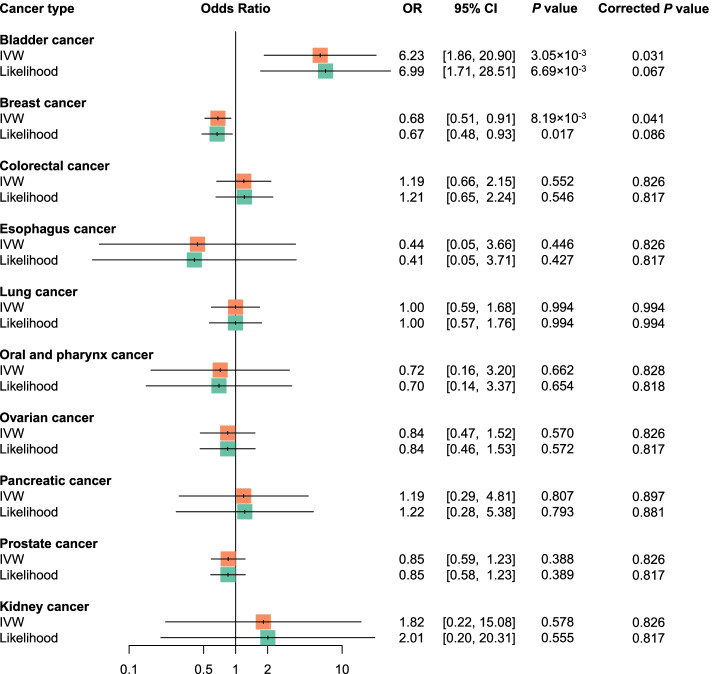
Forest plots of univariable Mendelian randomization (MR) estimates between circulating vitamin E and cancer risk in cancer-specific GWAS. The odds ratio (OR) was estimated using inverse variance weighting (IVW) and likelihood-based methods. The corrected *P*-value was calculated with false discovery rate (FDR) method

Figure S2 shows MR estimates of circulating vitamin E and each cancer risk. For the univariable MR analyses shown in Fig. 1, circulating vitamin E was not associated with risk of eight cancers, including colorectal, esophagus, lung, oral and pharynx, ovarian, pancreatic, prostate, and kidney cancer, where all *P*-values were above 0.05 (*P*_IVW_ > 0.05, *P*_Likelihood_ > 0.05, *P*_GRS_ > 0.05, Additional file 1: Table S5). Notably, circulating vitamin E was causally associated with an increased risk of bladder cancer (OR_IVW_ = 6.23, *P*_IVW_ = 3.05×10^-3^; OR_Likelihood_ = 6.99, *P*_Likelihood_ = 6.69×10^-3^; OR_GRS_ = 7.34, *P*_GRS_ = 1.57×10^-3^), but a decreased risk of breast cancer (OR_IVW_ = 0.68, *P*_IVW_ = 8.19×10^-3^; OR_Likelihood_ = 0.67, *P*_Likelihood_ = 0.017). These associations remained borderline significant after accounting for multiple comparisons across ten cancers (bladder cancer: *P*_IVW_ = 0.031, *P*_Likelihood_ = 0.067; breast cancer: *P*_IVW_ = 0.041, *P*_Likelihood_ = 0.086).

### Sensitivity analysis for causal estimation across each cancer

There was no heterogeneity or directional pleiotropy for each causal estimation (*P*_heterogeneity_ > 0.05; Additional file 1: Fig. S2; *P*_MR-Egger intercept_ > 0.05; Additional file 1: Table S5). Besides, leave-one-out analysis did not identify any outlying instruments (Additional file 1: Table S6). When profiling the association of each IV and multiple traits, we found that rs964184 and rs11057830 were associated with a total of 24 traits under *P* < 5.00×10^-8^ (Additional file 1: Table S7). Therefore, we performed multivariable MR analysis to adjust for the influence of each confounding trait, that is, the effect acting in particular through these traits. The association of circulating vitamin E with breast cancer risk attenuated to non-significant, indicating that the effect was most likely mediated by lipid-related traits such as cholesterol and lipoprotein. However, bladder cancer retained a robust, potentially causal relationship with circulating vitamin E (almost all adjusted *P* < 0.05; Table 2).

**Table 2:** Multivariable Mendelian randomization (MR) analysis for the associations of circulating vitamin E with the risk of bladder cancer and breast cancer

Corrected trait	Bladder cancer			Breast cancer		
	Beta^a^	95% CI^a^	*P*^a^	Beta^a^	95% CI^a^	*P*^a^
E70-E90 metabolic disorders	3.03	0.69, 5.37	0.011	−0.18	−0.73, 0.36	0.507
E78 disorders of lipoprotein metabolism and other lipidemias	2.96	0.91, 5.01	4.70×10^-3^	−0.23	−0.70, 0.25	0.349
Eosinophill count	2.61	1.15, 4.07	4.53×10^-4^	−0.36	−0.71, −0.01	0.043
Eosinophill percentage	2.34	1.02, 3.66	5.28×10^-4^	−0.37	−0.69, −0.05	0.022
High cholesterol	2.99	1.16, 4.83	1.38×10^-3^	−0.28	−0.71, 0.15	0.204
High light scatter reticulocyte count	3.07	1.26, 4.89	8.93×10^-4^	−0.30	−0.73, 0.13	0.165
High light scatter reticulocyte percentage	2.94	1.22, 4.65	7.85×10^-4^	−0.31	−0.71, 0.10	0.137
I20-I25 ischemic heart diseases	1.58	−0.15, 3.32	0.073	−0.26	−0.66, 0.15	0.211
I25 chronic ischemic heart disease	1.62	−0.28, 3.51	0.095	−0.23	−0.67, 0.21	0.308
Lymphocyte count	1.29	−0.15, 2.74	0.080	−0.34	−0.67, −0.01	0.046
Mean corpuscular hemoglobin	1.13	−0.70, 2.95	0.225	−0.28	−0.70, 0.15	0.202
Mean corpuscular hemoglobin concentration	2.67	1.17, 4.17	4.67×10^-4^	−0.35	−0.71, 0.01	0.057
Mean corpuscular volume	2.83	0.34, 5.32	0.026	−0.15	−0.73, 0.43	0.607
Mean platelet (thrombocyte) volume	2.66	1.05, 4.27	1.18×10^-3^	−0.29	−0.67, 0.08	0.126
Mean reticulocyte volume	3.09	1.19, 4.98	1.42×10^-3^	−0.28	−0.72, 0.17	0.223
Mean sphered cell volume	3.14	1.04, 5.23	3.29×10^-3^	−0.24	−0.72, 0.25	0.341
Monocyte count	1.81	0.60, 3.02	3.40×10^-3^	−0.38	−0.67, −0.10	0.008
Monocyte percentage	1.47	0.19, 2.74	0.025	−0.38	−0.67, −0.08	0.012
Platelet count	2.74	1.14, 4.34	7.81×10^-4^	−0.31	−0.69, 0.07	0.109
Platelet crit	2.71	1.17, 4.25	5.70×10^-4^	−0.33	−0.69, 0.04	0.080
Platelet distribution width	2.81	1.18, 4.43	7.21×10^-4^	−0.31	−0.70, 0.07	0.112
Red blood cell (erythrocyte) distribution width	2.51	1.09, 3.94	5.43×10^-4^	−0.34	−0.67, 0.00	0.051
Reticulocyte count	3.15	1.25, 5.05	1.17×10^-3^	−0.29	−0.74, 0.16	0.207
Reticulocyte percentage	3.01	1.23, 4.79	9.32×10^-4^	−0.30	−0.72, 0.12	0.164

^a^Multivariable inverse variance weighting (IVW) method

### Validation in the UK Biobank cohort

In the validation stage with the UK Biobank cohort, there was no evidence to support the associations between genetically predicted circulating vitamin E and the risk of ten cancers (all *P*_GRS_ > 0.05; Table 3). In particular, the positive association between circulating vitamin E and bladder cancer observed in GWAS was not replicated in this cohort (HR = 0.86, *P* = 0.918). Further random effects meta-analysis combining the GRS results for bladder cancer from GWAS and UK Biobank cohort still yielded a non-significant result (*I*^*2*^ = 45.8%, *P*_meta_ = 0.186). In the sensitivity analysis with incident and prevalent cancer cases, the association of circulating vitamin E with the risk of esophagus and kidney cancer became significant (*P* < 0.05), but they failed to survive the FDR correction (*P*_FDR_ > 0.05; Additional file 1: Table S8). In addition, the random effects meta-analysis combing the GRS results from GWAS and UK Biobank cohort yielded non-significant results for the two cancers (esophagus: *I*^*2*^ = 73.3%, *P*_meta_ = 0.607; kidney: *I*^*2*^ = 81.8%, *P*_meta_ = 0.540).

**Table 3:** Genetic risk score (GRS) analysis for the associations of vitamin E with cancer risk in the UK Biobank cohort

Cancer type	Cases	Method^a^	HR^b^	95% CI^b^	*P*^b^	Corrected *P*^c^
Bladder cancer	526	Circulating vitamin E-based GRS	0.86	0.05, 14.64	0.918	0.983
		One-sample weighted GRS	0.70	0.28, 1.75	0.444	0.989
		One-sample unweighted GRS	0.75	0.29, 1.93	0.551	0.988
Breast cancer	4350	Circulating vitamin E-based GRS	2.30	0.86, 6.14	0.096	0.337
		One-sample weighted GRS	1.06	0.82, 1.36	0.674	0.989
		One-sample unweighted GRS	1.06	0.82, 1.38	0.664	0.988
Colorectal cancer	2621	Circulating vitamin E-based GRS	0.43	0.12, 1.54	0.194	0.400
		One-sample weighted GRS	1.19	0.79, 1.80	0.404	0.989
		One-sample unweighted GRS	1.22	0.80, 1.87	0.348	0.988
Esophagus cancer	460	Circulating vitamin E-based GRS	14.06	0.72, 275.16	0.082	0.337
		One-sample weighted GRS	1.01	0.38, 2.69	0.989	0.989
		One-sample unweighted GRS	0.99	0.36, 2.72	0.988	0.988
Lung cancer	1700	Circulating vitamin E-based GRS	0.79	0.16, 3.89	0.772	0.983
		One-sample weighted GRS	1.17	0.70, 1.96	0.542	0.989
		One-sample unweighted GRS	1.16	0.69, 1.97	0.573	0.988
Oral and pharynx cancer	458	Circulating vitamin E-based GRS	1.03	0.05, 21.74	0.983	0.983
		One-sample weighted GRS	0.93	0.35, 2.50	0.891	0.989
		One-sample unweighted GRS	0.87	0.32, 2.39	0.792	0.988
Ovarian cancer	437	Circulating vitamin E-based GRS	2.09	0.09, 47.08	0.644	0.983
		One-sample weighted GRS	0.87	0.39, 1.92	0.724	0.989
		One-sample unweighted GRS	0.86	0.38, 1.96	0.722	0.988
Pancreatic cancer	506	Circulating vitamin E-based GRS	1.08	0.06, 20.08	0.960	0.983
		One-sample weighted GRS	0.94	0.37, 2.41	0.898	0.989
		One-sample unweighted GRS	0.96	0.37, 2.52	0.934	0.988
Prostate cancer	4882	Circulating vitamin E-based GRS	0.54	0.21, 1.39	0.200	0.400
		One-sample weighted GRS	0.93	0.72, 1.19	0.572	0.989
		One-sample unweighted GRS	0.97	0.75, 1.26	0.840	0.988
Kidney cancer	649	Circulating vitamin E-based GRS	0.11	0.01, 1.54	0.101	0.337
		One-sample weighted GRS	0.68	0.30, 1.57	0.370	0.989
		One-sample unweighted GRS	0.73	0.31, 1.70	0.466	0.988

^a^Circulating vitamin E-based GRS, derived from three circulating vitamin E-SNPs; one-sample weighted and unweighted GRS, derived from dietary vitamin E-SNPs (*P*-value ≤ 5×10^-5^)

^b^Adjusted for sex, age, study centers, body mass index (BMI), smoking status, drinking status, and first ten principal components when appropriate

^c^The corrected *P*-value was calculated with false discovery rate (FDR) method

Subsequently, we performed the genome-wide analysis to identify variants associated with dietary vitamin E intake, but no SNPs were found beyond genome-wide significance threshold (*P* ≤ 5×10^-8^; Additional file 1: Fig. S3). Based on the suggestive significance threshold (*P* ≤ 5×10^-7^), we identified three significant variants (rs11889555 on 2q32.2, beta = 0.02, *P* = 7.59×10^-8^; rs139695510 on 13q32.1, beta = -0.03, *P* = 1.29×10^-7^; and rs12165526 on 22q13.31, beta = 0.03, *P* = 2.79×10^-7^) in all population, one significant variant (rs11889555 on 2q32.2, beta = 0.03, *P* = 4.33×10^-7^) in males, and one significant variant (rs201524387 on 13q21.1, beta = 0.03, *P* = 1.96×10^-7^; Additional file 1: Table S9) in females. Further, we annotated these loci with functional activity and cis-eQTL analysis. Interestingly, rs11889555 had a high function score and significantly affected the expression of multiple nearby genes in blood samples (Additional file 1: Table S10).

For the one-sample MR analysis of dietary vitamin E intake, in the first-stage model, the weighed and unweighted vitamin E associated GRSs with a threshold of *P*-value ≤ 5.00×10^-5^ showed the strongest correlation with observed vitamin E level and were then used for predicting dietary vitamin E in the second-stage model (Additional file 1: Table S11). We found that the genetically predicted dietary vitamin E intake was not significantly associated with the risk of all ten cancers (*P*_FDR_ of weighted and unweighted GRS > 0.05; Table 3), consistent with findings of sensitivity analysis (Additional file 1: Table S8).

## Discussion

In this large-scale genetic association study, we evaluated the causal relationship of circulating vitamin E with the risk of ten common cancers capitalizing on the largest available cancer-specific GWAS data and UK Biobank cohort of European ancestry. Our current MR study, despite its largely augmented sample size and strong instruments, did not reveal convincing evidence to support causal associations of genetically predicted circulating vitamin E and dietary vitamin E intake with the risk of ten cancers.

Previous observational epidemiological studies have reported associations between vitamin E intake and the risk of these cancers [4, 32–42], and part of our results were supported by these reports. A previous meta-analysis including 24 studies suggested an inverse association between plasma α-tocopherol and breast cancer risk, but the association was not significant in the European population [41]. de Munter et al found that intake of dietary vitamin E did not support a protective association with oral and pharynx cancer risk using Netherlands cohort study data with 120,852 participants [33]. A systematic review including prospective cohort studies with over 200 ovarian cancer cases (*n* = 24) did not find a significant association between vitamin E concentrations and the risk of ovarian cancer [34]. Another cohort study including 10 studies in North America and Europe with 501,857 women also indicated that intakes of vitamins A, C, and E were not significantly associated with ovarian cancer risk [35]. Besides, the association of vitamin E supplementation with the risk of prostate cancer was not found in the Physicians’ Health Study II randomized trial with 14,641 men [5].

In addition, multiple observational studies have reported significant associations between vitamin E and a decreased risk of esophagus, colorectal, lung, pancreatic, kidney, and bladder cancer [36–40, 43]. For instance, a meta-analysis with 6431 subjects found that colorectal cancer patients had lower concentrations of serum vitamin E compared to healthy controls, especially in European populations [36]. A recent prospective study with 22,781 Finnish male smokers reported a 24% significant reduction in the risk of lung cancer for the fifth quintile compared with the bottom quartile of baseline α-tocopherol concentration [37]. A meta-analysis of 10 studies with 2976 patients and 254,393 controls observed a 13% lower risk of pancreatic cancer for the highest compared with the lowest level of vitamin E intake among European populations [38]. Cui et al. found an inverse relationship between dietary vitamin E intake and the risk of esophagus cancer among European and non-European populations using meta-analysis including 14 studies with 3013 cases and 11,384 non-cases [39]. Shang et al. reported a significant reduction in the risk of renal cell carcinoma for the highest intake compared with the lowest intakes of vitamin E concentrations among European populations using meta-analysis including 7 studies with 5789 cases and 14,866 controls [40]. A recent meta-analysis with 575,601 participants from the USA and Europe indicated that vitamin E consumption was inversely associated with the risk of bladder cancer; Chen et al. also found that α-tocopherol, the main isoform of vitamin E, was associated with a decreased risk of bladder cancer [42, 43]. However, our MR analysis with sufficient power did not support the associations between circulating vitamin E and the risk of above six cancers, indicating that the results of observational studies may need to be validated in further studies.

Vitamin E is a group of fat-soluble antioxidant nutrients consisting of eight natural isoforms. All isoforms scavenge reactive oxygen species through the presence of phenolic hydrogen in their chromanol ring [44]. Oxidative stress has been demonstrated to be involved in the pathogenesis of multiple diseases, especially for cancer. Oxidative stress can lead to free radical chain reaction causing lipid peroxidation, but vitamin E plays a vital role in breaking the free radical chain reaction, preventing lipid peroxidation, and protecting biological membrane [45, 46]. Therefore, the anticancer activity of vitamin E has been studied extensively. However, our findings of this MR study indicated that increasing circulating vitamin E concentrations or vitamin E intake was unlikely to result in clinical benefit for reducing cancer risk, which provided an important public health message that vitamin E supplementation may not be useful for cancer prevention.

Our study has several strengths. This was the first large-scale MR analysis that systemically evaluated a causal association between circulating vitamin E and the risk of multiple cancers, leveraging cancer-specific GWAS data of 602,435 solid cancer cases and controls, and a validation in UK Biobank cohort of 355,543 individuals, the largest study of its kind. In addition, this MR analysis was performed with no signs of violation to MR assumptions, as tested by MR-Egger and median-based approaches. We performed multiple MR analyses based on individuals of European descent, largely reducing population stratification.

Several limitations also need to be acknowledged. The main challenge with this study is the limited availability of genetic instruments for circulating vitamin E concentrations, with only 3 genetic variants explaining 1.7% of variation. This has implications for the detection of pleiotropy using MR Egger—although none of our pleiotropy tests reveals statistically significant violations, these diagnostic analyses are likely to be underpowered; therefore, more IVs related to circulating vitamin E and dietary vitamin E need to be identified. In addition, the 3 IVs were only associated with α-tocopherol levels, and we need to consider the effects of other isoforms of tocopherol and tocotrienol (e.g., γ- and δ-tocopherols) on cancer risk.

## Conclusions

In summary, this is the first largest MR study making causal inferences between circulating vitamin E concentrations and the risk of multiple cancers among European population. Our MR does not convincingly support a causal effect of vitamin E on the risk of cancer development, which delivers an important public health message that administration of vitamin E supplementation may not be necessary for prevention of cancers. Nevertheless, further studies are warranted to validate such findings as well as to demonstrate causal associations across ancestries.

## Supplementary Information

**Additional file 1: Table S1.** Summary of cancer-specific GWAS data included in Mendelian randomization (MR) analysis. **Table S2.** ICD-10 diagnosis codes for ten cancers in the UK Biobank cohort. **Table S3.** Characteristics of circulating vitamin E-associated instrumental variants (IVs). **Table S4.** Associations of circulating vitamin E instrumental variants (IVs) with multiple cancer risk in cancer-specific GWAS. **Table S5.** Mendelian randomization (MR) analysis for the associations of circulating vitamin E with cancer risk in cancer-specific GWAS. **Table S6.** Leave-one-out analysis for the associations between circulating vitamin E and cancer risk in cancer-specific GWAS. **Table S7.** Summary of 24 traits related to circulating vitamin E associated SNPs. **Table S8.** Sensitivity analysis of genetic risk score (GRS) analysis for the associations of vitamin E with cancer risk in the UK Biobank cohort. **Table S9.** Summary of dietary vitamin E intake associated SNPs derived from the UK Biobank cohort. **Table S10.** Functional annotation of dietary vitamin E intake associated SNPs. **Table S11.** Summary of dietary vitamin E associated variants in the UK Biobank cohort. **Figure S1.** Conceptual framework of Mendelian randomization (MR) analysis in this study. **Figure S2.** The effect of each variant on circulating vitamin E (exposure) and cancer risk (outcome) in cancer-specific GWAS. **Figure S3.** Manhattan and quantile-quantile (QQ) plots for the dietary vitamin E intake-related GWASs in the UK Biobank cohort.

## Data Availability

All data generated or analyzed during this study are included in this published article and its supplementary information files or available from the corresponding author upon reasonable request.

## Abbreviations

BMI: Body mass index
CI: Confidence interval
eQTL: Expression quantitative trait loci
GRS: Genetic risk score
GWAS: Genome-wide association study
HR: Hazard ratio
HWE: Hardy-Weinberg equilibrium
IV: Instrumental variable
IVW: Inverse variance weighting
MAF: Minor allele frequency
MR: Mendelian randomization
OR: Odds ratio
SD: Standard deviation
SELECT: Selenium and Vitamin E Cancer Prevention Trial
SNP: Single-nucleotide polymorphism
