# Correction: Association between circulating vitamin E and ten common cancers: evidence from large-scale Mendelian randomization analysis and a longitudinal cohort study

**DOI:** 10.1186/s12916-022-02493-z

**Published:** 2022-07-29

**Authors:** Junyi Xin, Xia Jiang, Shuai Ben, Qianyu Yuan, Li Su, Zhengdong Zhang, David C. Christiani, Mulong Du, Meilin Wang

**Affiliations:** 1 Jiangsu Cancer Hospital, Jiangsu Institute of Cancer Research, The Affiliated Cancer Hospital of Nanjing Medical University, Nanjing, China; 2 Department of Environmental Genomics, Jiangsu Key Laboratory of Cancer Biomarkers, Prevention and Treatment, Collaborative Innovation Center for Cancer Personalized Medicine, Center for Global Health, School of Public Health, Nanjing Medical University, 101 Longmian Avenue, Jiangning District, Nanjing, 211166 China; 3 Department of Clinical Neuroscience, Center for Molecular Medicine, Karolinska Institutet, Stockholm, Sweden; 4 Department of Environmental Health, Harvard T.H. Chan School of Public Health, Boston, MA USA; 5 Department of Medicine, Massachusetts General Hospital, Boston, MA USA; 6 Department of Biostatistics, Center for Global Health, School of Public Health, Nanjing Medical University, 101 Longmian Avenue, Jiangning District, Nanjing, 211166 China; 7 The Affiliated Suzhou Hospital of Nanjing Medical University, Suzhou Municipal Hospital, Gusu School, Nanjing Medical University, Suzhou, China

**Correction: BMC Med 20, 168 (2022)**

**https://doi.org/10.1186/s12916-022-02366-5**

The original article [1] contained an error whereby the production team handling the article mistakenly omitted Meilin Wang as a co-corresponding author. This omission has since been fixed.
