# Supplementary material for: Association between circulating vitamin E and ten common cancers: evidence from large-scale Mendelian randomization analysis and a longitudinal cohort study
Source: BMC Med. 2022 May 11;20:168. doi: 10.1186/s12916-022-02366-5 (PMC9092790; doi:10.1186/s12916-022-02366-5)
Supplement: Supplementary file 1 — Additional file 1: Table S1. Summary of cancer-specific GWAS data included in Mendelian randomization (MR) analysis. Table S2. ICD-10 diagnosis codes for ten cancers in the UK Biobank cohort. Table S3. Characteristics of circulating vitamin E-associated instrumental variants (IVs). Table S4. Associations of circulating vitamin E instrumental variants (IVs) with multiple cancer risk in cancer-specific GWAS. Table S5. Mendelian randomization (MR) analysis for the associations of circulating vitamin E with cancer risk in cancer-specific GWAS. Table S6. Leave-one-out analysis for the associations between circulating vitamin E and cancer risk in cancer-specific GWAS. Table S7. Summary of 24 traits related to circulating vitamin E associated SNPs. Table S8. Sensitivity analysis of genetic risk score (GRS) analysis for the associations of vitamin E with cancer risk in the UK Biobank cohort. Table S9. Summary of dietary vitamin E intake associated SNPs derived from the UK Biobank cohort. Table S10. Functional annotation of dietary vitamin E intake associated SNPs. Table S11. Summary of dietary vitamin E associated variants in the UK Biobank cohort. Figure S1. Conceptual framework of Mendelian randomization (MR) analysis in this study. Figure S2. The effect of each variant on circulating vitamin E (exposure) and cancer risk (outcome) in cancer-specific GWAS. Figure S3. Manhattan and quantile-quantile (QQ) plots for the dietary vitamin E intake-related GWASs in the UK Biobank cohort. [file 12916_2022_2366_MOESM1_ESM.pdf]

Supplementary information

Table S1. Summary of cancer-specific GWAS data included in Mendelian randomization (MR) analysis.

Cancer type	Sample size (N = 602,435)		Data type	Source[a]
	Cases (N = 297,699)	Controls (N = 304,736)		
Bladder cancer	5,930	5,468	Individual-level data	dbGaP (phs000346.v2.p2)
Breast cancer	122,977	105,974	Summary-level data	BCAC
Colorectal cancer	24,476	23,073	Individual-level data	dbGaP (phs001078.v1.p1, phs001315.v1.p1, phs001415.v1.p1)
Esophagus cancer	2,268	1,865	Individual-level data	dbGaP (phs000869.v1.p1)
Lung cancer	29,266	56,450	Summary-level data	TRICL-ILCCO OncoArray data
Oral and pharynx cancer	4,950	2,907	Individual-level data	dbGaP (phs001202.v1.p1)
Ovarian cancer	22,406	40,941	Summary-level data	OCAC
Pancreatic cancer	4,970	3,532	Individual-level data	dbGaP (phs000206.v5.p3)
Prostate cancer	79,148	61,106	Summary-level data	PRACTICAL
Kidney cancer	1,308	3,420	Individual-level data	dbGaP (phs000351.v1.p1)

[a] dbGaP, the database of Genotypes and Phenotypes; BCAC, the Breast Cancer Association Consortium (http://bcac.ccge.medschl.cam.ac.uk/); OCAC, the Ovarian Cancer Association Consortium (http://ocac.ccge.medschl.cam.ac.uk/); PRACTICAL, the Prostate Cancer Association Group to Investigate Cancer Associated Alterations in the Genome (http://practical.icr.ac.uk/blog/); TRICL-ILCCO, the Transdisciplinary Research in Cancer of the lung and the International Lung Cancer Consortium.

Table S2. ICD-10 diagnosis codes for ten cancers in the UK Biobank cohort.

Cancer type	Cases	ICD-10 codes
Bladder cancer	526	C67
Breast cancer (female)	4,350	C50
Colorectal cancer	2,621	C18, C19, C20
Esophagus cancer	460	C15
Lung cancer	1,700	C33, C34
Oral and pharynx cancer	458	C00-C14
Ovarian cancer (female)	437	C56
Pancreatic cancer	506	C25
Prostate cancer (male)	4,882	C61
Kidney cancer	649	C64

Table S3. Characteristics of circulating vitamin E-associated instrumental variants (IVs).

Chr	SNP	Position	Nearby gene	Allele[a]	Beta[b]	SE[b]	*P*[b]
11	rs964184	116648917	*ZNF259*	C/G	0.04	0.01	7.80×10^{-12}
12	rs11057830	125307053	*SCARB1*	G/A	0.03	0.01	8.20×10^{-9}
19	rs2108622	15990431	*CYP4F2*	C/T	0.03	0.01	1.40×10^{-10}

[a] Reference/effect allele.

[b] Obtained from previous Vitamin E GWAS among European ancestry, Beta and SE are based on per effect allele per unit change in log vitamin E level.

Table S4. Associations of circulating vitamin E instrumental variants (IVs) with multiple cancer risk in cancer-specific GWAS.

Cancer type	SNP	Allele[a]	OR[b]	95% CI[b]	*P*[b]
Bladder cancer	rs964184	C/G	1.02	0.94, 1.10	0.665
	rs11057830	G/A	1.10	1.02, 1.19	0.015
	rs2108622	C/T	1.08	1.02, 1.14	0.013
Breast cancer	rs964184	C/G	0.98	0.96, 1.00	0.043
	rs11057830	G/A	0.98	0.97, 1.00	0.089
	rs2108622	C/T	0.99	0.98, 1.01	0.351
Colorectal cancer	rs964184	C/G	1.02	0.98, 1.05	0.395
	rs11057830	G/A	1.02	0.98, 1.06	0.351
	rs2108622	C/T	0.99	0.96, 1.02	0.532
Esophagus cancer	rs964184	C/G	1.07	0.93, 1.22	0.361
	rs11057830	G/A	0.93	0.81, 1.06	0.266
	rs2108622	C/T	0.94	0.85, 1.04	0.202
Lung cancer	rs964184	C/G	1.02	0.99, 1.06	0.207
	rs11057830	G/A	1.01	0.97, 1.04	0.708
	rs2108622	C/T	0.98	0.96, 1.00	0.112
Oral and pharynx cancer	rs964184	C/G	0.95	0.86, 1.05	0.285
	rs11057830	G/A	0.97	0.89, 1.07	0.560
	rs2108622	C/T	1.03	0.96, 1.11	0.411
Ovarian cancer	rs964184	C/G	0.99	0.96, 1.03	0.684
	rs11057830	G/A	0.99	0.96, 1.03	0.685
	rs2108622	C/T	1.00	0.97, 1.03	0.843
Pancreatic cancer	rs964184	C/G	0.95	0.87, 1.04	0.283
	rs11057830	G/A	0.99	0.91, 1.08	0.868
	rs2108622	C/T	1.06	0.99, 1.13	0.104
Prostate cancer	rs964184	C/G	0.99	0.96, 1.01	0.255
	rs11057830	G/A	1.00	0.98, 1.03	0.725
	rs2108622	C/T	1.00	0.98, 1.01	0.631
Kidney cancer	rs964184	C/G	1.03	0.90, 1.18	0.651
	rs11057830	G/A	1.12	0.99, 1.27	0.080
	rs2108622	C/T	0.95	0.85, 1.05	0.305

[a] Reference/effect allele.

[b] Adjusted for sex, age and first 10 principal components for individual level GWAS data (bladder cancer, colorectal cancer, esophagus cancer, oral and pharynx cancer, pancreatic cancer and kidney cancer).

Table S5. Mendelian randomization (MR) analysis for the associations of circulating vitamin E with cancer risk in cancer-specific GWAS.

Cancer type	Method[a]	Estimate[b]	95% CI[b]	*P*
Bladder cancer	IVW	6.23	1.86, 20.90	3.05×10^{-3}
	Likelihood	6.99	1.71, 28.51	6.69×10^{-3}
	GRS	7.34	2.13, 25.24	1.57×10^{-3}
	Egger (intercept)	0.279	-0.018, 0.576	0.066
Breast cancer	IVW	0.68	0.51, 0.91	8.19×10^{-3}
	Likelihood	0.67	0.48, 0.93	0.017
	Egger (intercept)	0.015	-0.054, 0.084	0.676
Colorectal cancer	IVW	1.19	0.66, 2.15	0.552
	Likelihood	1.21	0.65, 2.24	0.546
	GRS	1.19	0.66, 2.15	0.568
	Egger (intercept)	-0.045	-0.206, 0.116	0.584
Esophagus cancer	IVW	0.44	0.05, 3.66	0.446
	Likelihood	0.41	0.05, 3.71	0.427
	GRS	0.48	0.05, 4.31	0.508
	Egger (intercept)	-0.47	-0.995, 0.054	0.079
Lung cancer	IVW	1.00	0.59, 1.68	0.994
	Likelihood	1.00	0.57, 1.76	0.994
	Egger (intercept)	-0.107	-0.267, 0.053	0.190
Oral and pharynx cancer	IVW	0.72	0.16, 3.20	0.662
	Likelihood	0.70	0.14, 3.37	0.654
	GRS	0.80	0.18, 3.61	0.771
	Egger (intercept)	0.192	-0.180, 0.564	0.311
Ovarian cancer	IVW	0.84	0.47, 1.52	0.570
	Likelihood	0.84	0.46, 1.53	0.572
	Egger (intercept)	0.005	-0.140, 0.149	0.951
Pancreatic cancer	IVW	1.19	0.29, 4.81	0.807
	Likelihood	1.22	0.28, 5.38	0.793
	GRS	1.35	0.33, 5.61	0.679
	Egger (intercept)	0.276	-0.117, 0.668	0.168
Prostate cancer	IVW	0.85	0.59, 1.23	0.388
	Likelihood	0.85	0.58, 1.23	0.389
	Egger (intercept)	0.035	-0.055, 0.124	0.447
Kidney cancer	IVW	1.82	0.22, 15.08	0.578
	Likelihood	2.01	0.20, 20.31	0.555
	GRS	1.98	0.24, 16.02	0.522
	Egger (intercept)	-0.036	-1.073, 1.002	0.947

[a] IVW, inverse variance weighting; GRS, genetic risk score method.

[b] OR value of IVW, likelihood and GRS methods; beta value of Egger intercept test.

Table S6. Leave-one-out analysis for the associations between circulating vitamin E and cancer risk in cancer-specific GWAS.

Cancer type	SNP (left out)	OR[a]	95% CI[a]	*P*[a]
Bladder cancer	rs964184	15.62	3.29, 74.07	5.39×10^{-4}
	rs11057830	4.22	1.06, 16.73	0.041
	rs2108622	4.14	0.89, 19.17	0.069
Breast cancer	rs964184	0.72	0.50, 1.04	0.079
	rs11057830	0.71	0.52, 0.98	0.036
	rs2108622	0.61	0.43, 0.88	8.36×10^{-3}
Colorectal cancer	rs964184	1.03	0.48, 2.19	0.939
	rs11057830	1.06	0.54, 2.07	0.864
	rs2108622	1.60	0.76, 3.37	0.215
Esophagus cancer	rs964184	0.10	0.01, 1.46	0.092
	rs11057830	0.71	0.06, 7.82	0.781
	rs2108622	1.09	0.07, 17.00	0.951
Lung cancer	rs964184	0.70	0.35, 1.37	0.294
	rs11057830	0.94	0.52, 1.70	0.839
	rs2108622	1.52	0.78, 2.96	0.215
Oral and pharynx cancer	rs964184	1.30	0.20, 8.55	0.787
	rs11057830	0.86	0.15, 4.87	0.867
	rs2108622	0.31	0.05, 2.10	0.232
Ovarian cancer	rs964184	0.86	0.40, 1.83	0.688
	rs11057830	0.86	0.44, 1.69	0.668
	rs2108622	0.80	0.38, 1.71	0.570
Pancreatic cancer	rs964184	2.92	0.49, 17.44	0.241
	rs11057830	1.35	0.27, 6.68	0.711
	rs2108622	0.42	0.07, 2.48	0.342
Prostate cancer	rs964184	0.96	0.59, 1.54	0.860
	rs11057830	0.78	0.52, 1.18	0.249
	rs2108622	0.84	0.53, 1.34	0.473
Kidney cancer	rs964184	1.62	0.11, 24.45	0.727
	rs11057830	0.63	0.05, 7.22	0.707
	rs2108622	6.99	0.50, 96.84	0.147

[a] IVW, inverse variance weighting method.

Table S7. Summary of 24 traits related to circulating vitamin E associated SNPs.

SNP	Effect allele	Trait	Beta[a]	*P*[a]
rs964184	C	E70-E90 Metabolic disorders	-8.65×10^{-3}	9.09×10^{-22}
rs964184	C	E78 Disorders of lipoprotein metabolism and other lipidaemias	-9.58×10^{-3}	2.45×10^{-31}
rs964184	C	Eosinophill count	2.43×10^{-3}	2.03×10^{-13}
rs964184	C	Eosinophill percentage	0.03	6.99×10^{-13}
rs964184	C	High cholesterol	-0.02	1.87×10^{-103}
rs964184	C	High light scatter reticulocyte count	-2.50×10^{-4}	4.98×10^{-26}
rs964184	C	High light scatter reticulocyte percentage	-5.46×10^{-3}	1.94×10^{-25}
rs964184	C	Mean corpuscular haemoglobin concentration	-0.03	2.15×10^{-27}
rs964184	C	Mean corpuscular volume	0.09	6.17×10^{-18}
rs964184	C	Mean platelet (thrombocyte) volume	-0.03	9.84×10^{-38}
rs964184	C	Mean reticulocyte volume	0.28	1.01×10^{-46}
rs964184	C	Mean sphered cell volume	0.19	2.50×10^{-52}
rs964184	C	Monocyte count	2.40×10^{-3}	6.39×10^{-9}
rs964184	C	Platelet count	1.71	3.46×10^{-37}
rs964184	C	Platelet crit	8.24×10^{-4}	1.28×10^{-13}
rs964184	C	Platelet distribution width	-0.03	9.26×10^{-160}
rs964184	C	Red blood cell (erythrocyte) distribution width	0.03	3.30×10^{-26}
rs964184	C	Reticulocyte count	-7.70×10^{-4}	1.60×10^{-32}
rs964184	C	Reticulocyte percentage	-0.02	9.38×10^{-32}
rs11057830	A	E70-E90 Metabolic disorders	5.16×10^{-3}	5.31×10^{-9}
rs11057830	A	E78 Disorders of lipoprotein metabolism and other lipidaemias	4.71×10^{-3}	4.91×10^{-9}
rs11057830	A	High cholesterol	5.39×10^{-3}	3.89×10^{-9}
rs11057830	A	I20-I25 Ischaemic heart diseases	4.44×10^{-3}	2.75×10^{-9}
rs11057830	A	I25 Chronic ischaemic heart disease	4.10×10^{-3}	2.46×10^{-9}
rs11057830	A	Lymphocyte count	-8.75×10^{-3}	3.65×10^{-8}
rs11057830	A	Mean corpuscular haemoglobin	-0.03	9.68×10^{-10}
rs11057830	A	Mean corpuscular volume	-0.07	1.55×10^{-10}
rs11057830	A	Mean sphered cell volume	-0.08	2.41×10^{-10}
rs11057830	A	Monocyte count	2.62×10^{-3}	1.09×10^{-10}
rs11057830	A	Monocyte percentage	0.05	5.93×10^{-22}

[a] From Gene ATLAS database (http://geneatlas.roslin.ed.ac.uk/) ($P < 5\times10^{-8}$).

Table S8. Sensitivity analysis of genetic risk score (GRS) analysis for the associations of vitamin E with cancer risk in the UK Biobank cohort.

Method	Cancer type	Cases	Method[a]	OR/HR[b]	95% CI[b]	*P*[b]	Corrected *P*[c]
Analysis with incident and prevalent cancer cases in case-control design	Bladder cancer	1,035	Circulating vitamin E based GRS	0.75	0.10, 5.74	0.781	0.887
			One-sample weighted GRS	0.47	0.25, 0.91	0.026	0.260
			One-sample unweighted GRS	0.52	0.26, 1.01	0.053	0.530
	Breast cancer	10,913	Circulating vitamin E based GRS	1.27	0.65, 2.48	0.477	0.887
			One-sample weighted GRS	1.14	0.97, 1.35	0.122	0.610
			One-sample unweighted GRS	1.13	0.95, 1.35	0.168	0.560
	Colorectal cancer	4,660	Circulating vitamin E based GRS	1.13	0.43, 2.96	0.808	0.887
			One-sample weighted GRS	1.24	0.90, 1.69	0.184	0.613
			One-sample unweighted GRS	1.25	0.91, 1.72	0.167	0.560
	Esophagus cancer	626	Circulating vitamin E based GRS	13.45	1.03, 175.2	0.047	0.235
			One-sample weighted GRS	1.11	0.47, 2.57	0.816	0.925
			One-sample unweighted GRS	1.08	0.45, 2.57	0.861	0.961
	Lung cancer	2,155	Circulating vitamin E based GRS	1.25	0.30, 5.13	0.759	0.887
			One-sample weighted GRS	1.22	0.77, 1.93	0.393	0.768
			One-sample unweighted GRS	1.21	0.76, 1.93	0.425	0.708
	Oral and pharynx cancer	906	Circulating vitamin E based GRS	1.17	0.13, 10.43	0.886	0.887
			One-sample weighted GRS	0.73	0.36, 1.48	0.386	0.768
			One-sample unweighted GRS	0.67	0.32, 1.37	0.267	0.668
	Ovarian cancer	965	Circulating vitamin E based GRS	2.62	0.32, 21.44	0.369	0.887
			One-sample weighted GRS	0.82	0.48, 1.40	0.461	0.768
			One-sample unweighted GRS	0.78	0.45, 1.37	0.389	0.708
	Pancreatic cancer	601	Circulating vitamin E based GRS	0.82	0.06, 12.11	0.887	0.887
			One-sample weighted GRS	1.04	0.44, 2.47	0.925	0.925

			One-sample unweighted GRS	1.08	0.45, 2.62	0.865	0.961
	Prostate cancer	7,743	Circulating vitamin E based GRS	0.59	0.28, 1.27	0.179	0.597
			One-sample weighted GRS	1.02	0.83, 1.25	0.869	0.925
			One-sample unweighted GRS	1.04	0.84, 1.28	0.727	0.961
	Kidney cancer	1,077	Circulating vitamin E based GRS	0.06	0.01, 0.46	0.007	0.070
			One-sample weighted GRS	0.94	0.49, 1.78	0.84	0.925
			One-sample unweighted GRS	1	0.52, 1.94	0.995	0.995
Additionally adjusting for socioeconomic and chronic disease status	Bladder cancer	526	Circulating vitamin E based GRS	0.98	0.06, 16.7	0.991	0.991
			One-sample weighted GRS	0.69	0.27, 1.75	0.437	0.939
			One-sample unweighted GRS	0.74	0.28, 1.90	0.527	0.931
	Breast cancer	4,350	Circulating vitamin E based GRS	2.31	0.86, 6.18	0.096	0.350
			One-sample weighted GRS	1.04	0.81, 1.34	0.758	0.939
			One-sample unweighted GRS	1.04	0.80, 1.35	0.786	0.931
	Colorectal cancer	2,621	Circulating vitamin E based GRS	0.38	0.10, 1.38	0.14	0.350
			One-sample weighted GRS	1.19	0.78, 1.80	0.416	0.939
			One-sample unweighted GRS	1.22	0.80, 1.87	0.363	0.931
	Esophagus cancer	460	Circulating vitamin E based GRS	10.22	0.51, 203.11	0.127	0.350
			One-sample weighted GRS	0.96	0.36, 2.59	0.939	0.939
			One-sample unweighted GRS	0.95	0.35, 2.62	0.923	0.931
	Lung cancer	1,700	Circulating vitamin E based GRS	0.76	0.15, 3.80	0.743	0.991
			One-sample weighted GRS	1.29	0.77, 2.17	0.334	0.939
			One-sample unweighted GRS	1.25	0.74, 2.12	0.409	0.931
	Oral and pharynx cancer	458	Circulating vitamin E based GRS	0.89	0.04, 18.92	0.939	0.991
			One-sample weighted GRS	0.89	0.33, 2.40	0.818	0.939
			One-sample unweighted GRS	0.83	0.30, 2.28	0.713	0.931
	Ovarian cancer	437	Circulating vitamin E based GRS	1.42	0.06, 32.79	0.826	0.991

		One-sample weighted GRS	0.84	0.38, 1.87	0.669	0.939
		One-sample unweighted GRS	0.84	0.37, 1.92	0.679	0.931
Pancreatic cancer	506	Circulating vitamin E based GRS	1.16	0.06, 21.8	0.92	0.991
		One-sample weighted GRS	0.93	0.36, 2.40	0.879	0.939
		One-sample unweighted GRS	0.96	0.36, 2.53	0.931	0.931
Prostate cancer	4,882	Circulating vitamin E based GRS	0.57	0.22, 1.46	0.237	0.474
		One-sample weighted GRS	0.92	0.71, 1.18	0.51	0.939
		One-sample unweighted GRS	0.95	0.74, 1.24	0.72	0.931
Kidney cancer	649	Circulating vitamin E based GRS	0.11	0.01, 1.53	0.101	0.350
		One-sample weighted GRS	0.70	0.30, 1.60	0.396	0.939
		One-sample unweighted GRS	0.74	0.32, 1.74	0.491	0.931

[a] Circulating vitamin E based GRS, derived from three circulating vitamin E-SNPs; one-sample weighted and unweighted GRS, derived from dietary vitamin E-SNPs (*P*-value $\leq 5\times10^{-5}$).

[b] Adjusted for sex, age, study centers, body mass index (BMI), smoking status, drinking status, and first ten principal components when appropriate.

[c] The corrected *P*-value was calculated with false discovery rate (FDR) method.

Table S9. Summary of dietary vitamin E intake associated SNPs derived from the UK Biobank cohort.

Population	Chr	SNP	Position[a]	Allele[b]	Beta[c]	95% CI[c]	*P*[c]
All	2	rs11889555	191886543	A/G	0.02	0.01, 0.03	7.59E-08
	13	rs139695510	97280551	T/A	-0.03	-0.05, -0.02	1.29E-07
	22	rs12165526	44361713	T/A	0.03	0.02, 0.05	2.79E-07
Male	2	rs11889555	191886543	A/G	0.03	0.02, 0.04	4.33E-07
Female	13	rs201524387	59189714	CT/C	0.03	0.02, 0.05	1.96E-07

[a] Chromosomal position, hg19/GRCh37 build.

[b] Major/minor allele.

[c] Adjusted for sex, age, study centers, body mass index (BMI), smoking status, drinking status, and first ten principal components when appropriate.

Table S10. Functional annotation of dietary vitamin E intake associated SNPs.

Population	Locus	SNP	Nearby gene	Promoter histone marks	Enhancer histone marks	DNAse	Proteins bound	Motifs changed	eGenes[a]
All	2q32.2	rs11889555	STAT1	8 organs	19 organs	BLD, BLD	POL2	4 altered motifs	GLS, TMEM194B, HIBCH, STAT1, INPP1
	13q32.1	rs139695510	HS6ST3					Mef2	
	22q13.31	rs12165526	SAMM50					6 altered motifs	
Male	2q32.2	rs11889555	STAT1	8 organs	19 organs	BLD,BLD	POL2	4 altered motifs	GLS, TMEM194B, HIBCH, STAT1, INPP1
Female	13q21.1	rs201524387	RNY4P29					11 altered motifs	

[a] Significant genes with cis-eQTL effects, derived from eQTLGen database.

Table S11. Summary of dietary vitamin E associated variants in the UK Biobank cohort.

Population	Threshold of *P*-value	nSNPs	Weighted GRS				Unweighted GRS			
			R^2	F statistic	Beta[a]	*P*[a]	R^2	F statistic	Beta[a]	*P*[a]
All	$5×10^{-7}$	3	0.002	86.75	1	$6.74×10^{-20}$	0.002	83.28	0.03	$3.34×10^{-19}$
	$5×10^{-6}$	9	0.004	222.61	1	$3.70×10^{-49}$	0.004	212.67	0.02	$3.69×10^{-47}$
	$5×10^{-5}$	69	0.025	1274.96	0.98	$1.30×10^{-278}$	0.024	1206.63	0.02	$9.65×10^{-264}$
Male	$5×10^{-7}$	1	0.001	24.76	0.03	$4.33×10^{-7}$	0.001	24.76	0.03	$4.33×10^{-7}$
	$5×10^{-6}$	8	0.008	181.03	1	$3.30×10^{-41}$	0.007	170.46	0.03	$5.93×10^{-39}$
	$5×10^{-5}$	44	0.034	823.96	0.97	$1.84×10^{-179}$	0.032	771.55	0.03	$5.62×10^{-168}$
Female	$5×10^{-7}$	1	0.001	27.94	0.03	$1.96×10^{-7}$	0.001	27.94	0.03	$1.96×10^{-7}$
	$5×10^{-6}$	6	0.005	139.3	0.99	$1.57×10^{-31}$	0.005	130.87	0.03	$7.56×10^{-30}$
	$5×10^{-5}$	69	0.045	1234.95	0.94	$1.56×10^{-266}$	0.042	1146.41	0.02	$1.62×10^{-248}$

[a] Adjusted for sex, age, study centers, body mass index (BMI), smoking status, drinking status, and first 10 principal components when appropriate.

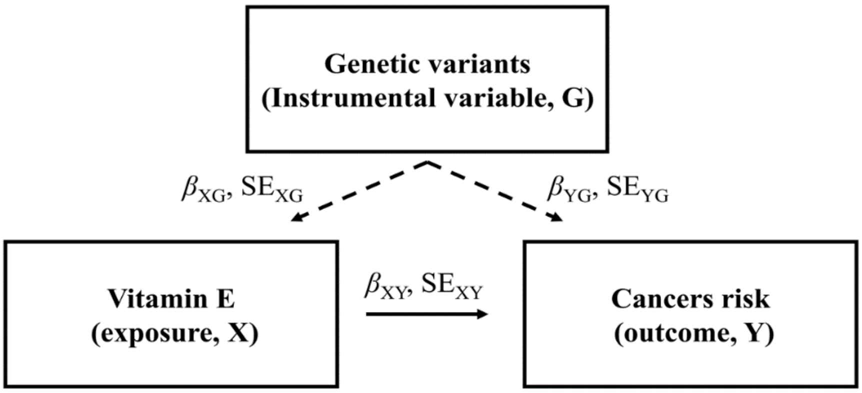

Figure S1. Conceptual framework of Mendelian randomization (MR) analysis in this study. We aim to estimate the unbiased causal relationship between vitamin E and cancer risk by incorporating genetic variants as instrumental variables (IVs). The association between vitamin E and cancer risk (β_{XY}, SE_{XY}) is estimated using the association of IVs (i.e., SNP) with vitamin E (β_{XG}, SE_{XG}) and cancer risk (β_{YG}, SE_{YG}).

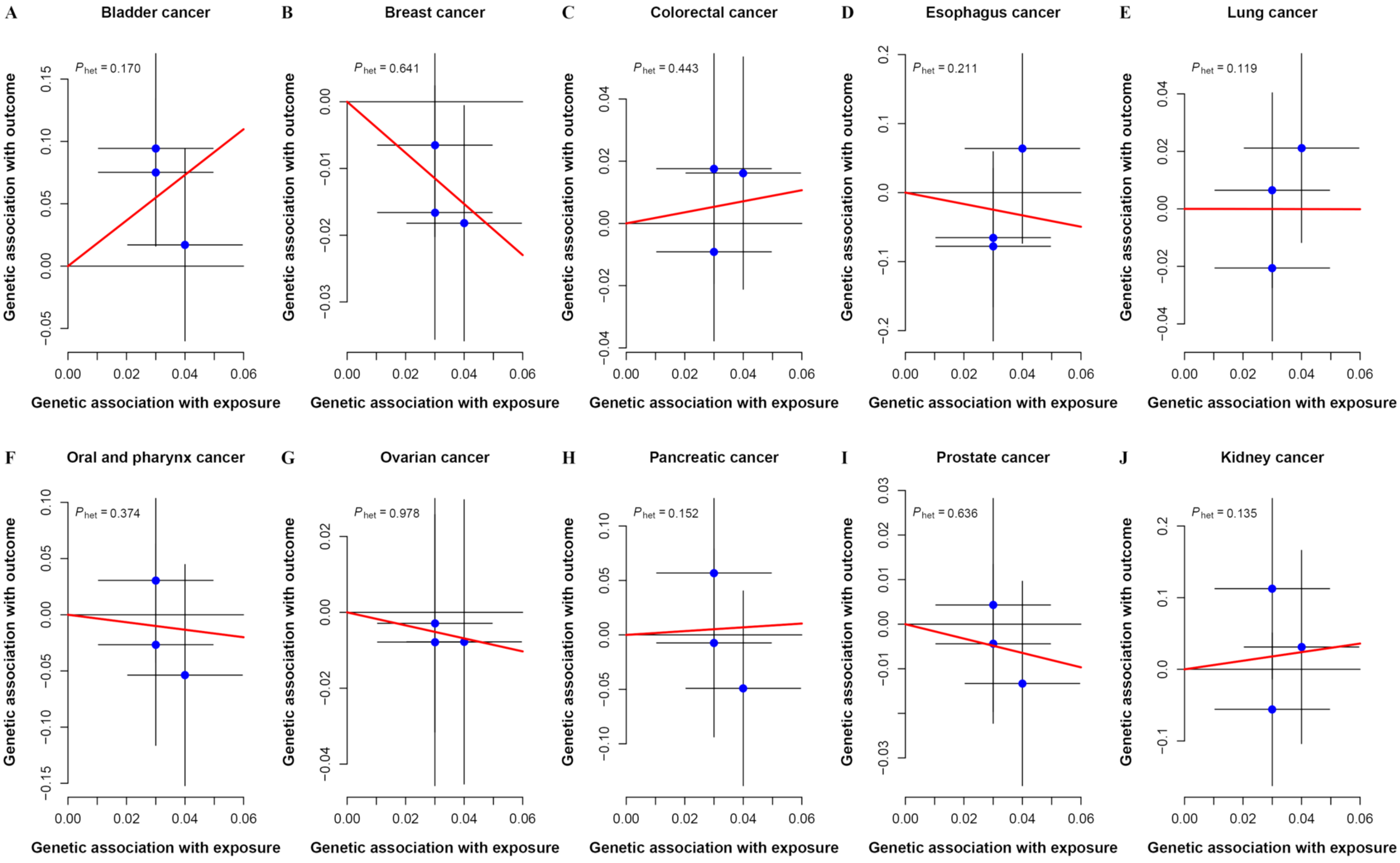
A
Bladder cancer
P_{het} = 0.170
B
Breast cancer
P_{het} = 0.641
C
Colorectal cancer
P_{het} = 0.443
D
Esophagus cancer
P_{het} = 0.211
E
Lung cancer
P_{het} = 0.119
F
Oral and pharynx cancer
P_{het} = 0.374
G
Ovarian cancer
P_{het} = 0.978
H
Pancreatic cancer
P_{het} = 0.152
I
Prostate cancer
P_{het} = 0.636
J
Kidney cancer
P_{het} = 0.135
Genetic association with outcome
Genetic association with exposure
0.00
0.02
0.04
0.06

Figure S2. The effect of each variant on circulating vitamin E (exposure) and cancer risk (outcome) in cancer-specific GWAS. Error bars around each SNP are 95% confidence intervals. A best fit regression line (dashed line) was plotted using the inverse variance weighting (IVW) based estimate. P_{het} was calculated using heterogeneity test. (A) Bladder cancer; (B) breast cancer; (C) colorectal cancer; (D) esophagus cancer; (E) lung cancer; (F) oral and pharynx cancer; (G) ovarian cancer; (H) pancreatic cancer; (I) prostate cancer; (J) kidney cancer.

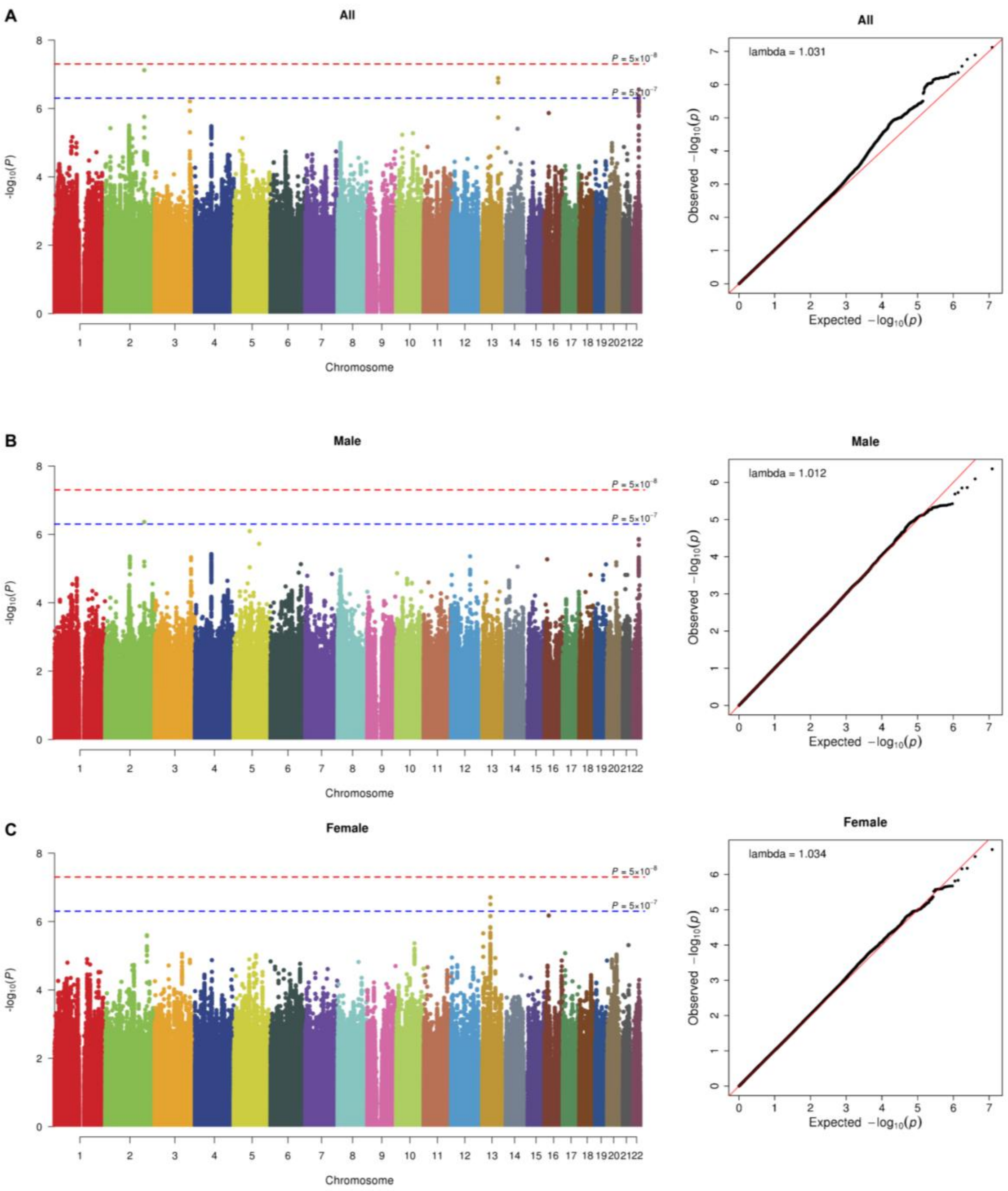

Figure S3. Manhattan and quantile-quantile (QQ) plots for the dietary vitamin E intake-related GWASs in the UK Biobank cohort. The associations (-log10 (*P*-value) values, Y-axis) are plotted against genomic position (X-axis by chromosome and the chromosomal position of NCBI build 37). The red dashed line indicates the genome-wide significance threshold ($P=5\times10^{-8}$). The blue dashed line indicates the suggestive significance threshold ($P=5\times10^{-7}$). (A) All population; (B) male population and (C) female population.
